# Cardiac Peptides—Current Physiology, Pathophysiology, Biochemistry, Molecular Biology, and Clinical Application

**DOI:** 10.3390/biology11020330

**Published:** 2022-02-18

**Authors:** Toshio Nishikimi, Johji Kato

**Affiliations:** 1 Department of Cardiovascular Medicine, Kyoto University Graduate School of Medicine, 54 Kawahara-cho, Shogoin, Sakyo-ku, Kyoto 606-8507, Japan; 2 Department of Medicine, Wakakusa Tatsuma Rehabilitation Hospital, 1580 Ooaza Tatsuma, Daito City 574-0012, Osaka, Japan; 3 Frontier Science Research Center, Faculty of Medicine, University of Miyazaki, Kiyotake, Miyazaki 889-1692, Japan; jkjpn@med.miyazaki-u.ac.jp; 4 Cardiovascular Medicine, University of Miyazaki Hospital, Kiyotake, Miyazaki 889-1692, Japan

The heart has long been considered a pumping organ, consisting of muscles. Therefore, when it was discovered that the heart secretes hormones, scientists and doctors around the world received this fact with great surprise. About 40 years have passed since the atrial natriuretic peptide (ANP), the first cardiac peptide, was discovered [1]. Subsequently, the brain natriuretic peptide (BNP) and C-type natriuretic peptide (CNP) were discovered, and natriuretic peptide receptor-A (NPR-A), NPR-B, and NPR-C receptors were also identified [2]. Because the guanylate cyclase domain is not coupled to NPR-C, it has long been recognized as a clearance receptor; however, recent studies have shown that Gi protein is coupled to NPR-C and that it exerts various biological effects [3]. A new ligand for NPR-C, osteocrin, was also discovered from bone [4], and a recent study showed that osteocrin had an important role in suppressing the progression of heart failure by inhibiting NPR-C [5]. Plasma BNP level is increased according to the severity of heart failure, and guidelines worldwide recommend use of BNP in the diagnosis of heart failure. In addition, recombinant ANP and BNP are effective in improving the hemodynamics of heart failure and have been used in the treatment of acute decompensated heart failure [6]. Recently, an angiotensin receptor and neprilysin inhibitor (ARNI) has been found to be more effective in treating chronic heart failure patients compared with an angiotensin-converting enzyme inhibitor [7]. ANP and CNP are well degraded by neprilysin, while the substrate specificity of BNP is low [8]. Whether BNP can be used as a biomarker for heart failure when using ARNI is currently being debated. 

Endothelin was discovered in 1988 as a potent vasoconstriction and pressor peptide isolated from the culture supernatant of porcine aortic endothelial cells [9]. Many studies have shown that endogenous endothelin is involved in the progression of various cardiovascular diseases, and as a result, a variety of endothelin antagonists have been developed [10]. Several studies showed that experimentally, endothelin antagonist is a very effective drug in the treatment of heart failure, but clinical trials have failed to improve mortality or heart failure hospitalization rates in heart failure patients [10]. However, endothelin receptor antagonists have been shown to be effective in treating pulmonary arterial hypertension, and current guidelines approved the use of endothelin antagonists in the treatment of pulmonary arterial hypertension [11].

Adrenomedullin is a potent vasodilator peptide which consists of 52 amino acids, originally discovered in human pheochromocytoma tissue [12]. Subsequent studies showed adrenomedullin was highly expressed in heart tissue, and adrenomedullin has the inhibitory effect of proliferation and collagen production in fibroblasts and cardiac hypertrophy in myocytes, and it also has a positive inotropic effect [13]. Highly expressed cardiac adrenomedullin in heart failure may regulate cardiac function and cardiac hypertrophy [13]. In addition, several studies showed that the intravenous administration of adrenomedullin to patients with heart failure improved hemodynamics in heart failure [14]. The adrenomedullin receptor is formed by a complex of seven-transmembrane calcitonin receptor-like receptor (CRL) and single-transmembrane receptor activity modifying protein (RAMP) 2, and the type of RAMP forms its ligand selectivity. For example, RAMP2/CRL and RAMP3/CRL are receptors for adrenomedullin and adrenomedullin 2, while RAMP1/CLR forms a receptor for the calcitonin gene-related peptide. When cardiac myocyte-specific RAMP2 deletion was induced, mice exhibited dilated cardiomyopathy-like heart failure with cardiac dilatation and myofibril disruption, supporting the hypothesis that increased cardiac adrenomedullin in heart failure may compensate for a failing heart [15].

Ghrelin was discovered in the stomach of rats in 1999 as an endogenous ligand for growth hormone secretagogues receptor (GHS-R) [16]. Ghrelin exerts its potent growth-hormone-releasing and orexigenic activities by binding to specific receptors in the hypothalamus. However, subsequent studies demonstrated that ghrelin has a cardiovascular function [17]. Indeed, the chronic administration of ghrelin improved cardiac function and left ventricular remodeling in a rat model of chronic heart failure induced by myocardial infarction. Moreover, the long-term administration of ghrelin improves LV function, exercise capacity, and muscle wastage in patients with heart failure [18].

Cholecystokinin (CCK), a gut–brain peptide, is expressed at the mRNA and protein levels in both atrial and ventricular myocytes. Interestingly, the post-translational processing of proCCK in myocytes is substantially different from intestinal and cerebral CCK peptides. The analysis of extracts of porcine cardiac tissue by specific proCCK radioimmunoassays showed that cardiac proCCK expression shifted from the right atrium in newborn piglets to include the left atrium in adolescent pigs [19]. The plasma proCCK level is increased during exercise in parallel with proBNP. Furthermore, the plasma proCCK level—but not the CCK level—is increased in severe heart failure patients.

Apelin is an endogenous peptide ligand for the APJ receptor, which is widely expressed in the human body. The apelin/APJ system exerts various physiological functions, including vasodilation, inotropic effects, heart development, the control of fluid homeostasis, and obesity. Both apelin and angiotensin II are substrates for angiotensin-converting enzyme 2 (ACE2), which degrades peptides and thus negatively regulates their agonistic activities. In addition, endogenous apelin negatively regulates the renin–angiotensin system via the upregulation of ACE2 [20]. Furthermore, a second ligand for the APJ receptor, elabela, was identified as an essential hormone for heart development, and it has been reported to have physiological effects similar to apelin [21]. As apelin has both inotropic and cardioprotective effects in heart failure, further research regarding apelin/elabela systems may encourage therapeutic applications of these peptides.

For a long time, the heart has been considered to be an organ that pumps blood to the whole body. However, the discovery of ANP also revealed that the heart has the property of an endocrine organ, and subsequent studies demonstrated that the heart secretes a lot of cardiac peptides. Thus, the mammalian heart is by now an established endocrine organ, which secretes a variety of cardiac peptides. The heart not only secretes various cardiac peptides but is also a target organ expressing receptors for these cardiac peptides. It has been elucidated that these cardiac peptides play an important role in the pathophysiology of heart failure, and several of them indeed have been clinically applied. In addition, new findings regarding cardiac peptides are still being reported. The presentation of ongoing research regarding cardiac peptides is one of the main objectives of this Special Issue of *Biology*. I look forward to receiving your research and review articles.
